# Stigmatization of men who have sex with men in health care settings in East Africa is based more on perceived gender role-inappropriate mannerisms than having sex with men

**DOI:** 10.1080/16549716.2020.1816526

**Published:** 2020-09-28

**Authors:** Michael W. Ross, John Kashiha, Lucy R. Mgopa

**Affiliations:** a Program in Human Sexuality, Department of Family Medicine and Community Health, University of Minnesota Medical School, Minneapolis, MN, USA; b CHESA, Dar es Salaam, Tanzania; c Department of Psychiatry, Muhimbili University of Health Sciences, Dar Es Salaam, Tanzania

**Keywords:** Health discrimination, gay, bisexual, gender role, Africa

## Abstract

**Background**: Healthcare Workers may stigmatize and discriminate against Men who have Sex with Men in East Africa.

**Objective**: To understand the predictors of abuse and discrimination of sexual minority men in healthcare settings by Healthcare workers in seven cities in Tanzania.

**Method**: In total, 300 sexual minority men over the age of 18 were interviewed in 7 Tanzanian cities by trained local interviewers. Abuse from others (physical, verbal, sexual, discrimination/humiliation), and abuse from Healthcare workers, was ascertained. Gender role mannerisms were self-rated by the respondent, and at the end of the interview, by the interviewer, on a Likert scale from very feminine to very masculine. Respondents also indicated whether they had revealed their homosexual behavior or had it exposed in the health consultation.

**Results**: Median age was 27. Verbal abuse and community discrimination were the most commonly reported forms of abuse. Eighty-four percent had visited a healthcare center with a sexually related complaint (usually a sexually transmitted infection); of these, 24% reported abuse or discrimination from from a healthcare worker. Correlation between self-rated gender role mannerisms and interviewer-rated was r = 0.84. Regression analysis indicated that the degree of perceived gender role nonconformity was the major and significant predictor from Healthcare worker abuse: confirmation of homosexual behavior was non-significant. Gender role nonconformity predicted 21% of the variance in health worker abuse.

**Conclusion**: There is speculation that abuse of sexual minority men by Healthcare workers in public clinics is due to factors in addition to their sexual behavior as gay/bisexual, and that it is due to violating perceived gender roles. Data confirm that perceived feminine gender role is a significant predictor, of abuse in healthcare and other settings. Common confusion between homosexual behavior and gender role norms may trigger discrimination, which may be as much due to violation of perceived gender roles as having sex with other men.

## Background

Stigmatization of men who have sex with men (MSM) in Sub-Saharan Africa (SSA), and specifically discrimination in health care settings, has been widely reported. Such discrimination or abuse may include shaming of MSM in clinics or refusal of treatment. MSM describe the difficulty of trying to balance this seesaw of visibility versus the need for access to treatment, of trying to remain invisible versus the need to obtain proper and timely treatment, and the need to provide false details (for example, on the sex of partner or site of infection with an STI) to try to obtain treatment. Treatment in the private sector is widely preferred due to the friendlier service, but for the vast majority of MSM, such clinics are unaffordable. Public clinics charge a fee but this is considerably lower than in private clinics.

In Tanzania, MSM behavior is criminalized with imprisonment up to life [1]: in 2007, 95% (95% CI ± 4%) of Tanzanian residents sampled in the Pew Global Attitudes Survey believed that homosexuality was not acceptable, and Tanzanian acceptance of homosexuality scores decreased between 2000–2003 and 2014–2017 [1,2]. Negative attitudes toward homosexuality in the health care sector probably reflect those in the general population.

In our own work in Tanzania [3], it appeared that in MSM who did not conform to the masculine gender stereotype in their dress or behavior were more visible, regardless of whether they were seeking treatment related to STI infection, or for broader health issues [4,5]. There were other suggestions in the literature that pointed in a similar direction. Magesa et al. [6] reported that stigmatization appeared to be higher among Tanzanian MSM with feminine mannerisms, who seemed to have the most negative clinic experiences. It may be that such individuals were less able to ‘pass’ as heterosexual. This is consistent with the finding of Sandfort et al. [7] in South Africa, who noted that men with physical appearance or attributes considered feminine were more likely to be discriminated against.

Moen et al. [8] also noted that in Tanzania, the term ‘gay’ is used to refer to men who assume behaviors or roles that are attributed to the female sex or men who take a receptive role in anal intercourse, while those who are insertive are considered regular ‘guys’ (conventionally masculine men), with versatile (both insertive and receptive) anal positioning far less common. They [9] describe in detail the historical and anthropological context and development of perceptions of MSM in Tanzania, with MSM falling into two main categories, based on sexual positioning. The data on sexual positioning are consistent with our Tanzanian data [3] that showed only 10% of MSM reported a versatile choice of position in anal sex.We use the terms gender role and gender norms as consistent with the UNICEF glossary of terms and concepts, referring to the social and cultural construct that distinguishes differences in the attributes of men and women (femininity and masculinity) in a particular cultural context [10]. UNICEF notes that the concept is ‘useful in analyzing how commonly shared practices legitimize discrepancies between sexes’ (p2).

African data are completely consistent with the classic work of Carrier [11,12] in Latino MSM, which noted that in many cultures, where there is clear social gender role demarcation, this gender role demarcation is also present in sexual practices between men, in which the person assuming the receptive role in anal intercourse is considered homosexual (the insertive partner may not be considered homosexual), and more feminine.

These data raised the question as to whether the relationship of stigma and discrimination against MSM which has been widely reported in SSA, is due to stigma associated with sex with other men (popularly considered ‘homosexuality’), to behavior perceived as gender role-inappropriate for men (feminine), or to some combination of these.

## Methods

Local research assistants, who were bilingual Kiswahili-English, trained community outreach workers, and members of a community-based organization (CBO) providing HIV and STI prevention services to key populations (CHESA: Community Health Education Services & Advocacy), interviewed 300 MSM over age 18 who had had sex with other men in the past 12 months.

Men were contacted and recruited through peer educator links with key populations, social media [13], phone call appointments, CBO databases, MSM networks and organizations working with MSM/gay men, CBO organizers, and snowball contacts. We previously characterized social networks in MSM in two cities in Tanzania and have demonstrated that there are clearly established social networks in Dar es Salaam, the major metropolitan area, and a smaller provincial city [14]. The respondents were interviewed in six of the ten largest cities in Tanzania, plus Iringa, a city in the southern highlands. This included most of the largest cities in Tanzania, including Dar es Salaam (pop. 4,364,541), Mwanza (706,543), Arusha (416,442), Mbeya (385,279), Tanga (273, 332), Unguja, Zanzibar (223,033), and Iringa (151,345) (2012 census populations).

Administration of the survey was conducted at agreed-upon safe locations where the respondent felt most comfortable and made the choice themselves, including bus stations, garages, workplaces, colleges or universities, and in cars. We moved to respondent-chosen locations given the political situation at the time of data collection in 2018, including condemnatory speech from politicians and negative media exposure. At the time of the survey administration, the research assistants explained the purpose of the research and the men gave verbal informed consent. No incentive was provided. Research took place in the first 8 months of 2018. Interviews took up to 30 minutes to complete but typically took about 15 minutes. Interviewers asked questions, clarified responses, and recorded interviewee information on the 21-item questionnaire, including both quantitative and open-ended responses, which was pre-tested on five members of the MSM community in Dar es Salaam.

All survey administration took place in Kiswahili or English, as the respondent preferred, with 98% being in Kiswahili. No details which might identify any respondent were recorded. Data were entered into SPSS and checked against the original paper interview copy during data cleaning.

Questionnaire: The questionnaire, which was initially pilot-tested on a sample of 5 MSM for clarity and sense, then further edited, collected data on age, education level, sexual identification (homosexual/gay, bisexual, heterosexual, transsexual/transgender, other), sexual activity with what sex over the past 5 years, how masculine or feminine the respondent perceived themselves (5-point Likert scale from Very feminine to Very masculine), whether the respondent had ever been the victim of physical violence or abuse, verbal abuse, discrimination or humiliation, sexual abuse, or abuse specifically from an HCW (Yes or No), whether the participant had visited an HCW because of a sexually or genitally related condition, whether the HCW was told (or found out) that the respondent had sex with other men, the types of abuse or discrimination encountered in a health care setting, and sexual activities engaged in over the past 12 months. Questions included ‘Have you been the victim of (physical violence or abuse)? Yes/No. *If Yes, by whom? Tick all that apply*: Family member; Partner; Neighbor; Person in street; Police; Co-worker; Health care worker; Sex worker/customer’. The type of violence or abuse, noted above, was, as separate subsequent questions: verbal abuse; discriminated against or humiliated; been a victim of sexual abuse. There was also a specific question, ‘Have you ever been a victim of abuse from a health care worker? (Yes/No)’.

Finally, the interviewer themselves rated how masculine or feminine the respondent appeared to the interviewer, based on observable characteristics including mannerisms, dress or speech (by checking a box at the end of the questionnaire against the same 5-point Likert scale from Very feminine to Very masculine) in order to assess the degree of respondent and external observer (interviewer) agreement.

Analysis: Data were entered into SPSS 25 for analysis. For demographic data, means and SDs were calculated for linear data, and frequencies and percentages for categorical data. Correlations between self-assessed and interviewer-assessed measures of social gender role (masculinity-femininity) were computed using Spearman correlations. Comparison of externally rated gender role by presence or absence of the types of abuse (physical, verbal, discrimination, sexual, health care), where 1 = Very feminine and 5 = Very masculine, was by *t*-test (separate variance assumed if F was significant at *p* <.05). Logistic regression (simultaneous entry) was carried out on the binary variable of health-care abuse, using interviewer-assessed gender role and the binary variable of telling (or the health care worker finding out) that the respondent was a man who had sex with other men. Interaction between the two predictors was included to create a saturated model. Because one in six of the sample reported that they were transsexual/transgender, and we believed that they might rate themselves as more highly feminine than an observer might, we used the observed external gender role rating of the interviewer in all analyses, believing that it would reflect more accurate observations of HCWs, coming from an external source. All significance levels were set at p < .05, two-tailed.

Patient and public involvement: No patients were involved. All participants were MSM recruited from public sites and organization websites. Results will be disseminated on the CBO websites used to recruit participants in a plain-language paragraph in Kiswahili and English, written by the second author.

## Results

**Figure 1.**
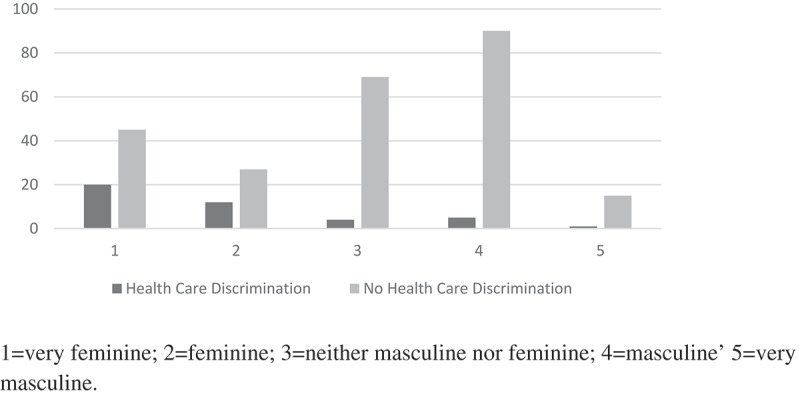
Externally rated gender role by presence of absence of health care discrimination.

Results are in Table 1 to 4 and Figure 1. The sample had a median age of 27, with median age of first sex with a man at age 12, and with a woman at age 15. Forty-five percent identified as gay/homosexual, 32% bisexual, 6% heterosexual, and 17% transsexual/transgender. Modal education was primary school (years 1–7: 39%) with a further 26% completing some secondary school (years 8–11 for ordinary secondary school and 12–13 for advanced secondary school). Abuse based on their sexuality was reported as physical abuse by 41%, verbal abuse by 63%, discrimination or humiliation by 59%, sexual abuse by 20%, and abuse by a health care worker 18%. A further 6% listed a discrimination in Table 2. Excluding health care abuse, 32.7% (n = 32.7) reported 0, 5.7% (n = 17) reported 1, 20.3% (n = 61) reported 2, 29.0% (n = 87) reported 3, and 12.3% (n = 37) reported all 4 of the four categories of abuse. Only one person (0.3%) reported health care abuse who had not also reported any other form of abuse. A large majority (84%, n = 254) had sought health care for a sexually related condition, and of those, only 21% (n = 53) indicated that they had disclosed or confirmed to the health care worker that they had sex with other men. Interviews were carried out in six of the major cities in Tanzania, with about a third (32.3%) coming from the major metropolis, Dar es Salaam (Arusha 11.3%, Iringa 11.0%, Mbeya 15.3%, Mwanza 13.7%, Tanga 9.0%, Unguja/Zanzibar 7.3%). The great majority of the interviews were in Kiswahili, with only five being conducted entirely in English. Correlation between self-rated gender role and externally (interviewer)-rated gender role was 0.84. There was a significant bivariate relationship between reported health care abuse and externally rated gender role (χ^2^ = 34.25, df = 4, p < .0005). The relationship between reported health care abuse and positioning in anal sex in the past 12 months (top, versatile, bottom) was not significant (χ^2 ^= 5.12, df = 3, p = .08).

**Table 1.** Demographic characteristics of sample^*^.

Age	Mean 29.0	Median 27.0	SD 7.34	Range 18–53	n = 300
Age of first sex with a man					
	Mean 13.01	Median 12.0	SD 3.80	Range 6–28	n = 170
Age of first sex with a woman					
	Mean 15.62	Median 15.0	SD 2.27	Range 8–23	n = 129
Sexual Orientation	n	%			
Gay/homosexual	136	45.3			
Bisexual	96	32.0			
Straight/Heterosexual	9	5.7			
Gender Identification					
Transsexual/Transgender	37	17.0			
Education					
None	3	1.0%			
Primary	116	38.7%			
Some Secondary School	80	26.37%			
Completed Secondary School	57	19.0%			
Some tertiary	10	3.3%			
University/College graduate	32	10.7%			
Abuse experiences					
Physical abuse	Yes	122	40.7		
	No	175	58.3		
Verbal abuse	Yes	189	63.0		
	No	107	35.7		
Discrimination/humiliation	Yes	177	59.0		
	No	121	40.3		
Sexual abuse	Yes	60	20.0		
	No	232	77.3		
Health care abuse	Yes	39	17.7		
	No	246	82.3%		
Sought health care for a Sexually-related condition	Yes	253	84.3%		
	No	47	15.7%		
If Yes, told Health Care Worker or confirmed that was MSM	Yes	53	24.1%		
	No	167	75.9%		
City of interview					
Arusha	34	11.3			
Dar es Salaam	97	32.3			
Iringa	33	11.0			
Mbeya	46	15.3			
Mwanza	41	13.7			
Tanga	27	9.0			
Unguja/Zanzibar	22	7.3			
Language of interview					
Kiswahili	291	English	5	Both	4

*Not all *n*’s sum to 300 or percentages sum to 100 because of missing values.

Table 2 indicates that the most common forms of discrimination were being verbally abused or threatened, followed by the refusal of services or a worse level of service than others there. Interviewers noted that ‘threats’ included being reported to police or being ejected from the clinic. This may also have been reported as verbal abuse in that category. On *t*-test, reported presence (*vs* reported absence) of almost all forms of abuse: physical abuse, verbal abuse, discrimination or humiliation, and abuse (or lower quality of care) by a health care worker, were significantly higher (p < .001) in those who were assessed externally as appearing more feminine. The exception was sexual abuse where there were no significant differences in externally perceived femininity (Table 3). Figure 1 illustrates the higher proportion of MSM with perceived non-normative gender role being stigmatized in a health care context.

**Table 2.** Discrimination encountered in health care*.

Form of discrimination	*n*
Given worse services than others there	14
Refused services	15
Abused verbally	23
Threatened	21
Other: Overcharged	4
Open-ended responses expanding quantitative responses:**	
Abusive language, harsh words, hate speech	3
Treatment denied or refused	3
Treatment delayed	14
Ignored	1
Sexually abused	2
Beaten	1
Exhibited, shown in more than one room	2
Slandered or false statements made	1
Unwelcome, bad reception	3
Despised	1
Talked down to (lectured)	1
Talked about (or reported: similar meaning, ‘kusemwa’ in Kiswahili) 7	

*More than one response could be given

**Translated from Kiswahili or in 6 cases, in English

**Table 3.** Differences by externally assessed gender role and forms of abuse.

Form of abuse		M	SD	*n*	*t*	df	*p*
Physical abuse	Yes	2.14	1.15	122	−9.41	244.20	.*000*
	No	3.37	1.05	175			
Verbal abuse	Yes	2.40	1.19	189	−10.58	276.17	.*000*
	No	3.67	0.87	107			
Discrimination	Yes	2.37	1.21	177	−9.95	294.46	.*000*
	No	3.59	0.89	121			
Sexual abuse	Yes	2.60	1.34	60	−1.73	85.80	.*09*
	No	2.93	1.22	232			
Health care worker	Yes	1.83	1.04	40	−5.89	283	.*000*
abuse	No	3.01	1.21	247			

Table 4 illustrates the logistic regression with the presence or absence of health care worker (HCW) abuse as the outcome, with being told or otherwise confirming that the respondent was MSM, and the externally rated gender role. The regression indicated, using the Nagelkerke pseudo-R^2^, that 22% of the variance in HCW abuse was accounted for by these two predictor variables, with externally assessed gender role being highly significant (p < 0.0001) and being exposed or identifying as an MSM to the HCW not significant as a predictor (p = 0.38). The interaction term was likewise not significant in the regression model.

**Table 4.** Regression of identification as homosexual and gender role on abuse by health care worker.

Variable	B	SE	Exp(B)	Wald	df	95% CI of Exp(B)	*p*
Told or confirmed to HCW that was MSM	−.78	.88	0.46	0.78	1	0.82–2.59	.38
Externally assessed gender role	.80	.22	2.22	13.72	1	1.46–3.39	.*000*
Interaction	.14	.44	1.15	0.10	1	0.49–2.73	.75
Constant	−0.75	.50	0.93	0.22	1		.88

Nagelkerke R^2^ = 0.22

## Discussion

These data were collected from a large sample of Tanzanian MSM in six different cities widely distributed across the country, who confirmed that they had had sex with another man in the past year. The comparison of externally rated gender role on a Very masculine to Very feminine Likert scale by the categories of discrimination showed that in every case excepting sexual abuse, there were very significant differences in experiencing abuse, with those externally rated as more feminine being abused physically, verbally, by discrimination, and by HCWs, more frequently than those externally rated as more masculine. These data indicate that gender role behavior (degree of masculinity or femininity expressed) was a significant predictor of abuse over most types of abuse, not just in health care. The data are consistent with our hypothesis that those with a perceived non-normative gender role (in this case, males with culturally consistent feminine mannerisms or appearance), are more likely to be discriminated against. However, we cannot know whether this is because of their perceived non-normative gender role as such, or because the person discriminating assumes, based on popular stereotype, that the man is homosexual.

Toska et al. [15] report that social protections can reduce sexual risk behavior in South African adolescents: receiving sensitive ‘care’ services from providers at sexual health care clinics was associated with lower rates of unprotected sex in adolescent girls but not boys. Other social protections, including school access and parental supervision, had additive effects in significantly reducing sexual risk behavior, particularly among girls. Clearly sensitive sexually related clinic care can play a major part in reducing HIV risk behavior. The lack of sensitive clinic care shown here (including refusing care or abusive lack of care) may significantly *increase* risk behavior and potential for disease transmission, through lack of treatment of STIs and the absence of safer sex counseling and encouragement. Clearly, further research in MSM populations in this area is needed.

The regression analysis (Table 4) utilizes data on whether the MSM is known to have sex with men by the HCW, and gender role as externally assessed by the interviewer. In this case, it is clear that the HCW discrimination was found to be predicted by the perceived feminine gender role, and that the MSM status was not a significant predictor. These data suggest that it may be the perception of a culturally non-normative (feminine) gender role, rather than the sexual behavior with another man (commonly referred to as homosexuality in Tanzania), which triggers a significant amount of the discrimination: in these data, the model accounts for more than a fifth of the variance of abuse in health care settings. The anecdotal suggestions in earlier research that perceived non-normative gender role may trigger discrimination, at least in clinical settings, appears to be confirmed. These data are also consistent with long-held anthropological observations in other cultures [11,12] and Tanzania [8,9] that the labelling of behavior as homosexual may be associated with those men who take a receptive role in anal intercourse, but that those taking the insertive role may not be so labelled. It would seem to be more the publically observed non-normative gender role, and not so much the sexual orientation or behavior, which activates a discriminatory reaction in some HCWs.

It could be speculated that the greater triggering power of gender role in discrimination may be due to a distinction between ‘doing’ and ‘being’ [16]. Sex with another man is a behavioral act that is not a visible marker of deviance and may be a transient and private situation (or if an insertive rather than receptive act, not so transgressive of male positioning). A perceived non-normative gender role may be a permanent and visible reminder that gender may be mutable, and the primacy of social, cultural and religious structures based on sex differences (as normatively perceived in a society) may be perceptibly and publically threatened. Our data further inform the literature which suggests that it is not the homosexual practices, as such, that are significantly associated with HCW abuse in MSM, but observation of a non-normative (feminine) gender role.

There are limitations in these data, collected in one country in East Africa, and in relatively low proportions of MSM who came out or were identified as having sex with other men by HCWs. However, this is likely to diminish the statistical significance rather than enhance it. Further, identification as MSM by an HCW was unevenly distributed across gender role, and more feminine appearing men may be more likely to be identified as MSM by HCWs. However, using external (interviewer-rated) rather than respondent self-rated gender role would also have reduced any respondent bias in rating. While Table 2 indicates the variety of forms of health service-related discrimination or abuse reported by the participants, we have few data indicating the level of abuse from HCWs perceived by the non-MSM population [17]. The fact that these data are on males further limits their generalizability to women who have sex with women, in SSA. What is considered a ‘gender-appropriate’ role or behavior is heavily culturally based, often including a religious component, and thus also limits generalizability.

These data are informative for understanding the source and motivation for discrimination, and especially HCW discrimination, against MSM. They suggest that discrimination is heavily linked to what are perceived as non-normative or ‘deviant’ gender roles in this society. Models to reduce discrimination in health care in MSM in SSA, such as the SPEND model [18], emphasize the importance of education of HCWs in sexual health and in understanding variation in human sexuality. While short-term workshops and sensitivity training to promote a better understanding of the complexities of interaction with and providing medical services to key populations are effective and important [19], they may attract those already interested in the area. General education in human sexuality for medical, nursing, midwifery, pharmacy and other health care professions may be a long-term proposition but will reach a larger population of health professionals. It may also have a greater impact on services and treatment for general sexual issues, including sexual dysfunction, pregnancy prevention, sexual violence, and other sexual health concerns across the entire population. If sexual orientation and gender role are conflated or confused, such education is based on false premises and is unlikely to be culturally responsive. Further, these data suggest that it will be more challenging to reduce discrimination against transgendered MSM, as the basis for that discrimination appears to go beyond sexual orientation (the sex of preferred sexual partner). However, these data from a Tanzanian MSM sample are suggestive and will require confirmation and extension in other SSA countries, and more research on the little-studied transgendered populations in the region [20].

## Paper context

Gay and bisexual men in East Africa reported receiving health care discrimination based on their sexual behavior, but these data suggest that MSM behavior may be a minor component of such stigmatization. Abuse against MSM from HCWs appears to be associated more with MSM’s perceived gender-inappropriate (feminine) behavior than MSM’s same-sex sexual behavior. Data highlight the need for HCW education on gender role and spectrum as well as on gay and bisexual behavior and identity for HCWs.
